# Reply to Comment on Huang, X., et al. “Sourdough Fermentation Degrades Wheat Alpha-Amylase/Trypsin Inhibitor (ATI) and Reduces Pro-Inflammatory Activity”. *Foods* 2020, *9*, 943

**DOI:** 10.3390/foods9101405

**Published:** 2020-10-03

**Authors:** Xin Huang, Michael Gänzle, Jussi Loponen, Detlef Schuppan

**Affiliations:** 1 Department of Food and Nutrition, Faculty of Agriculture and Forestry, University of Helsinki, PL 66, FI-00014 Helsinki, Finland; 2 Department of Agricultural, Food and Nutritional Science, University of Alberta, Edmonton, AB T6G 2P5, Canada; mgaenzle@ualberta.ca; 3 Fazer lab, Fazer Group, 01230 Vantaa, Finland; jussi.loponen@fazer.com; 4 Institute of Translational Immunology and Research Center for Immunotherapy, University Medical Center, Johannes Gutenberg University, 55131 Mainz, Germany; detlef.schuppan@unimedizin-mainz.de

We thank Dr. Laatikainen for the interest in our article and appreciate his comments. While some points raised are appreciated, there are several comments that we do not consider “on the point” or of relevance regarding our research report. 

**Sourdough modifies multiple bioactivities in wheat bread.** The main topic of our article was to investigate the changes of Alpha-Amylase/Trypsin Inhibitor (ATI) protein concentration and bioactivity in yeast-fermented bread vs. sourdough bread [1] rather than compare the impact of these two fermentations on the symptoms of irritable bowel syndrome (IBS) patients, as performed by Laatikainen et al. [2]. Laatikainen determined the fructan content in yeast bread (0.23 g per 100 g bread) vs. sourdough-fermented bread, finding a reduced fructan content in sourdough bread (0.06 per 100 g bread). While this was significant, the overall diet provided to the patients may otherwise not have had a reduced content of fermentable oligo-, di-, monosaccharides and polyols (FODMAPs) [2]. Thus the statement that the bread had a substantially reduced FODMAPs content that would then explain potential differences in patients’ complaints cannot be easily upheld. 

We also maintain that a comparison of sourdough bread to bread produced by a straight dough process may not enable to assess the contribution of specific wheat components, as sourdough fermentation has a profound impact on multiple wheat components that are known or suspected to contribute to symptoms in IBS or non-celiac wheat sensitivity (NCWS) patients. Sourdough fermentation reduces the FODMAP content of wheat [3,4], modifies and partially degrades gluten proteins and the gluten macropolymer [5], and partially degrades ATI [1]. In addition, sourdough bread also has a reduced content of phytate and can increase the content of dietary fibre by the synthesis of bacterial exopolysaccharides and by the modification of starch digestion, modifying bioactive phenolic compounds [6,7] and favouring beneficial changes in the gut microbiota [8]. A study in IBS patients comparing sourdough bread with yeast-fermented bread thus assesses the combined effect of all of these modifications [2]. A more targeted reduction in specific components without modifying of the bread making process is required to inform on the contribution of specific wheat components. This can be achieved, e.g., by using enzymes [4] or by using isogenic lactic acid bacteria that differ in specific metabolic properties [9].

**ATI contamination in gluten preparations.** Alpha-amylase/trypsin inhibitors (ATIs) that are water/salt-soluble and the CM ATIs are also chloroform/methanol-soluble. Commercial gluten isolate/vital gluten contains between 1 and 3 wt% (weight percent) of ATIs, depending on the starch separation and production process. Thus, a comparison of an ATI/gluten/free-diet and a commercial gluten containing diet (containing 3% of ATI) showed an intestinal and extra-intestinal immune activation in mouse models of disease that was equivalent to the same amount of purified ATIs in chow, whereas the same amount of gluten did not show immune activation, after the near complete removal of ATI by further extraction [10,11,12]. We performed several clinical studies to confirm the results of mouse studies in patients, including a study that confirmed disease activation in patients with familial Mediterranean fever [13].

**Dietary triggers of IBS and NCWS remain unknown.** While IBS patients appear to be a substantial subgroup of NCWS patients, another large subgroup of NCWS patients has mainly extra-intestinal symptoms or exacerbation of their chronic diseases [14]. The first appears to be due to an immediate reaction (atypical, IgE negative food allergy) to whole wheat, as assessed by endoscopic duodenal challenge and confocal laser endomicroscopy [15,16]. Here, wheat is the prominent food trigger; ATIs may contribute both as allergens and pro-inflammatory proteins to this novel form of atypical food allergy in patients with NCWS-IBS [10,11,12,17,18,19,20]. Therefore, in general, NCWS patients benefit from a gluten-free diet that is also an ATI-free diet and often a low-FODMAP diet. A FODMAP-reduced diet can improve IBS symptoms, mainly by reducing bloating, but is only sustainable in mild forms of IBS. Importantly, adverse effects of FODMAPs are dose-dependent and a supply of FODMAPs is important for a healthy gut microbiota. 

Certainly, more studies are needed to understand the role of different dietary triggers for NCWS and its subgroups of wheat-induced IBS (atypical wheat allergens) and wheat-induced exacerbation of chronic disease (ATIs). Here, the role of food processing in changing the immunogenicity of major (proteinaceous) triggers appears to play a major role, as exemplified by the wheat ATIs. Because IBS and NCWS have multiple dietary triggers, and because sourdough fermentation has a profound effect on the bioactive (inflammatory) components of wheat bread, future studies should include a thorough determination of biochemical modifications of proteins, carbohydrates and other wheat components as well as well-designed clinical studies. 
